# Retraction: Molecular docking and *in vivo*/*in vitro* studies of a novel thiadiazole Schiff base as a hepatoprotective drug against angiogenesis induced by breast cancer

**DOI:** 10.1039/d6ra90056a

**Published:** 2026-05-28

**Authors:** Norah F. Alqahtani, Mohammad Y. Alfaifi, Ali Shati A, Serag Eldin I. Elbehairi, Abdulrahman M. Saleh, Ebtesam S. Kotb, Waleed M. Serag, Reda F. M. Elshaarawy, Heba W. Alhamdi, Yasser A. Hassan

**Affiliations:** a Department of Chemistry, College of Science, University of Jeddah Jeddah 21589 Saudi Arabia; b King Khalid University, Faculty of Science, Biology Department Abha 9004 Saudi Arabia; c Tissue Culture and Cancer Biology Research Laboratory, King Khalid University Abha 9004 Saudi Arabia; d Cell Culture Lab, Egyptian Organization for Biological Products and Vaccines (VACSERA Holding Company) 51 Wezaret El-Zeraa St., Agouza Giza Egypt; e Department of Pharmaceutical Chemistry, Faculty of Pharmacy, Cairo University Kasr El-Aini Street Cairo 11562 Egypt; f Aweash El-Hagar Family Medicine Center, Epidemiological Surveillance Unit, MOHP Mansoura 35711 Egypt; g Department of Chemistry, Faculty of Science, Suez University 43533 Suez Egypt waleed.ibrahim@suezuniv.edu.eg; h Institut für Anorganische Chemie und Strukturchemie, Heinrich-Heine Universität Düsseldorf Düsseldorf Germany; i College of Sciences, Biology Department, King Khalid University Abha 61413 Saudi Arabia; j Department of Pharmaceutics, Faculty of Pharmacy, Delta University for Science and Technology Gamasa Egypt; k Department of Pharmaceutics and Pharmaceutical Technology, College of Pharmacy, Al-Kitab University Kirkuk Iraq

## Abstract

Retraction of ‘Molecular docking and *in vivo*/*in vitro* studies of a novel thiadiazole Schiff base as a hepatoprotective drug against angiogenesis induced by breast cancer’ by Norah F. Alqahtani *et al.*, *RSC Adv.*, 2024, **14**, 39027–39039, https://doi.org/10.1039/D4RA06398H.

The Royal Society of Chemistry, hereby wholly retracts this *RSC Advances* article due to concerns with the reliability of the data.

Concerns were raised regarding the Fig. 4 micrographs, which were previously published by the same authors in ref. 1, and the Fig. 10 flow cytometry plots, which were previously published in ref. 2 by different authors. The corresponding author has cooperated fully with our investigation and said that the duplication in Fig. 4 was due to the authors working on the same projects in parallel. They say that the wrong set of processed images were mistakenly inserted into the *RSC Advances* manuscript and have provided replacement images. The authors have acknowledged the issues with the Fig. 10 Flow Cytometry plots, and claim that these were outsourced to a third-party laboratory but have not been able to provide any evidence of this.

We attempted to inform all authors about the retraction of the article, but have we have not been able to reach Serag Eldin I. Elbehairi, Ebtesam S. Kotb, Abdulrahman M. Saleh, Ali Shati A, Mohammad Y. Alfaifi and Heba W. Alhamdi.

Waleed M. Serag has not indicated whether they agree with the decision to retract. The other authors have not responded.

Given the significance of these concerns, the Editor has lost confidence that the findings presented in this paper are reliable.

Signed: Laura Fisher, Executive Editor, *RSC Advances*

Date: 20th May 2026
